# Effect of Resident Physician Education Regarding Selective Chemoprophylaxis for the Prevention of Early Onset Group B Streptococcal Sepsis: An Outcome Study

**DOI:** 10.1155/S1064744994000116

**Published:** 1994

**Authors:** Jeffrey S. Greenspoon, Doron J. D. Rosen, Anita P. Sumen

**Affiliations:** 1 Department of Obstetrics and Gynecology Cedars-Sinai Medical Center Los Angeles CA USA; 2 Department of Obstetrics and Gynecology Room 1738 Cedars-Sinai Medical Center 8700 Beverly Boulevard Los Angeles CA 90048 USA

## Abstract

*Objective:* The aim of this study was to evaluate the effect of a voluntary protocol for selective intrapartum chemoprophylaxis on the incidence of early onset group B streptococcal sepsis (GBS EOS).

*Methods:* Cases of GBS EOS were defined as a positive GBS culture from a normally sterile fluid obtained during the first 7 days of life. All cases of GBS EOS at an urban, university-affiliated community hospital were reviewed during 2 time periods. The 2-year period before instituting a resident education program to promote selective chemoprophylaxis (1988–89) was retrospectively reviewed; the 2-year period after the education program was introduced (1990–91) was prospectively recorded. The outcome measure was the incidence of GBS EOS.

*Results:* The rate of GBS EOS was 7/14,335 deliveries (0.05%) before and 9/13,999 (0.064%) after the introduction of the education program (observed difference between proportions 0.016%, 95% confidence interval [CI] for the difference between the proportions –0.071% to 0.04%, *P* = not significant [NS]). The rate of GBS EOS in preterm infants was 5/1,331 (0.376%) before and 3/1,297 (0.23%) afterward (observed difference between proportions 0.14%, 95% CI –0.28% to 0.56%, *P* = NS). The incidence of GBS EOS did not decrease during the latter period due to failure of antepartum cultures to predict intrapartum GBS colonization (2 cases); non-compliance with voluntary recommendations to administer chemoprophylaxis (2 cases); failure of chemoprophylaxis or therapy for intraamniotic infection to prevent neonatal infection (3 cases); and occurrence of GBS EOS in infants without risk factors (2 cases).

*Conclusions:* An education program for resident physicians regarding chemoprophylaxis for GBS EOS did not significantly reduce the absolute incidence of disease. Alternative strategies are needed that redress the causes of failure inherent in the current guidelines. Some cases of GBS EOS are not preventable because the parturient does not have risk factors that indicate chemoprophylaxis.


Infectious Diseases in Obstetrics and Gynecology 1:210–215 (1994)
© 1994 Wiley-Liss, Inc.

### Effect of Resident Physician Education Regarding Selective Chemoprophylaxis for the Prevention of Early Onset Group B Streptococcal Sepsis: An Outcome Study

**Jeffrey S. Greenspoon, Doron J.D. Rosen, and Anita P. Sumen**

*Department of Obstetrics and Gynecology, Cedars-Sinai Medical Center, Los Angeles, CA*

Abstract

***Objective:*** The aim of this study was to evaluate the effect of a voluntary protocol for selective intrapartum chemoprophylaxis on the incidence of early onset group B streptococcal sepsis (GBS EOS).

***Methods:*** Cases of GBS EOS were defined as a positive GBS culture from a normally sterile fluid obtained during the first 7 days of life. All cases of GBS EOS at an urban, university-affiliated community hospital were reviewed during 2 time periods. The 2-year period before instituting a resident education program to promote selective chemoprophylaxis (1988–89) was retrospectively reviewed; the 2-year period after the education program was introduced (1990–91) was prospectively recorded. The outcome measure was the incidence of GBS EOS.

***Results:*** The rate of GBS EOS was 7/14,335 deliveries (0.05%) before and 9/13,999 (0.064%) after the introduction of the education program (observed difference between proportions 0.016%, 95% confidence interval [CI] for the difference between the proportions −0.071% to 0.04%, $P$ = not significant [NS]). The rate of GBS EOS in preterm infants was 5/1,331 (0.376%) before and 3/1,297 (0.23%) afterward (observed difference between proportions 0.14%, 95% CI −0.28% to 0.56%, $P$ = NS). The incidence of GBS EOS did not decrease during the latter period due to failure of antepartum cultures to predict intrapartum GBS colonization (2 cases); non-compliance with voluntary recommendations to administer chemoprophylaxis (2 cases); failure of chemoprophylaxis or therapy for intraamniotic infection to prevent neonatal infection (3 cases); and occurrence of GBS EOS in infants without risk factors (2 cases).

***Conclusions:*** An education program for resident physicians regarding chemoprophylaxis for GBS EOS did not significantly reduce the absolute incidence of disease. Alternative strategies are needed that redress the causes of failure inherent in the current guidelines. Some cases of GBS EOS are not preventable because the parturient does not have risk factors that indicate chemoprophylaxis.
© 1994 Wiley-Liss, Inc.

Key words

Pregnancy complications, infections, pregnancy outcome, outcome assessment (health care), neonate, septicemia

Early onset group B streptococcal sepsis (GBS EOS) is the most common single cause of neonatal bacterial infection.[1] In the past decade, the risk factors associated with GBS EOS were identified and the effectiveness of chemoprophylaxis during the intrapartum period was demonstrated.[2,3] Antepartum cultures are poor predictors of intrapartum colonization.[4,5] Furthermore, a reliable

Address correspondence/reprint requests to Dr. Jeffrey S. Greenspoon, Department of Obstetrics and Gynecology, Room 1738, Cedars-Sinai Medical Center, 8700 Beverly Boulevard, Los Angeles, CA 90048.

Presented at the 40th Annual Meeting of the American College of Obstetricians and Gynecologists, Las Vegas, NV, April 27–30, 1992.

Received December 9, 1993
Accepted March 15, 1994

Clinical Study rapid diagnostic test for group B *Streptococcus* (GBS) colonization that is feasible to use during the intrapartum period is not yet available.[4,6] Thus, it is not possible to identify patients colonized with GBS when they present in labor. Therefore, Minkoff and Mead[7] have recommended that all pregnant patients with preterm labor (PTL) or preterm premature rupture of membranes (PROM) whose GBS status is unknown receive chemoprophylaxis until a negative culture is obtained. Pregnant patients with preterm PROM and/or PTL are easily identified. They are at risk for GBS EOS and will benefit from chemoprophylaxis.[3] We assessed the absolute rate of GBS EOS before and after a specific resident education effort was begun at our institution.

### MATERIALS AND METHODS

This study was conducted at Cedars-Sinai Medical Center, Los Angeles, CA, an urban, university-affiliated, community medical center serving an ethnically mixed population. During the time interval from January 1, 1988, to December 31, 1989, there were no established management guidelines to prevent GBS EOS. From January 1, 1990, selective chemoprophylaxis was strongly recommended by the staff for patients with pregnancies complicated by PTL (<37 weeks) or preterm PROM. Term pregnancies complicated by prolonged ROM (P-ROM) (≥18 h) were also candidates for chemoprophylaxis, although this was left to the discretion of the physician.[7] Intrapartum fever which was thought to represent intraamniotic infection was an indication for antibiotic therapy with ampicillin and gentamicin or a therapeutically equivalent regimen.[8] Compliance with the chemoprophylactic regimen was voluntary. The resident education program consisted of a discussion of the risk factors for GBS EOS and the benefits of chemoprophylaxis during the daily work rounds whenever the management of a patient with preterm PROM and/or PTL was discussed with the resident staff. Chemoprophylaxis consisted of ampicillin, 2 g q 6 h. Patients with penicillin allergy received erythromycin.[7]

We reviewed all identifiable cases of GBS EOS to determine the incidence during both time periods. Obstetric data were reviewed to determine whether the incidence of GBS EOS decreased in patients with preterm delivery or preterm PROM. Cases of GBS EOS were identified by reviewing the ICDM-9 (International Classification of Diseases, Clinical Modification, 9th Revision) discharge diagnostic codes for GBS meningitis or GBS bacteremia. A computerized microbiologic database of all positive blood and cerebrospinal fluid cultures for GBS was reviewed to identify additional cases. Medical records were available for all the cases identified. Rates were compared using the program Confidence Interval Analysis.[9]

### RESULTS

During the 2-year period (1988–89) before the education program regarding GBS chemoprophylaxis, GBS EOS occurred in 7/14,335 deliveries (0.05%) compared with the 2-year period (1990–91) following the education program when there were 9/13,999 (0.064%) (observed difference between proportions 0.0155%, 95% confidence interval [CI] for the difference between the proportions −0.071% to 0.04%, $P$ = not significant [NS]). Among preterm deliveries fewer than 37 weeks gestation, there were 5/1,331 (0.376%) cases of GBS EOS before and 3/1,297 (0.23%) afterward (observed difference between proportions 0.14%, 95% CI −0.28% to 0.56%, $P$ = NS) (Table 1).

The overall incidence of GBS EOS did not differ between the 2 periods. The incidence of GBS EOS in PTL did not differ between the 2 time periods. However, if one considers cases delivered prior to 36 weeks gestation, then only 1 case of GBS EOS occurred in preterm infants during the latter time period when chemoprophylaxis was consistently recommended.

We did not determine the magnitude of compliance with chemoprophylaxis among patients with risk factors. Patients receiving expectant management for preterm PROM receive care in a special maternal-fetal medicine unit where the resident staff and perinatologist make rounds twice daily and review the management. We estimate that the compliance rate for chemoprophylaxis in this group approached 100%.

In the 1st study period (1988–89), 5 of the 7 cases were complicated by preterm PROM. One could prospectively anticipate a prolonged (≥18 h) time of ruptured membranes because expectant management was attempted in all these cases. Two additional cases of GBS EOS occurred in term

**TABLE 1. Incidence of GBS EOS before and after the introduction of a protocol for chemoprophylaxis**

| | Period before chemoprophylaxis (1988–89) | | Period after chemoprophylaxis (1990–91) | |
|---|---|---|---|---|
| | No. cases GBS EOS/No. deliveries | Rate | No. cases GBS EOS/No. deliveries | Rate |
| Preterm (<37 weeks gestation) | 5/1,331 | 1/266 (0.376%) | 3/1,297 | 1/432 (0.23%) |
| Term | 2/13,004 | 1/6,502 (0.015%) | 6/12,702 | 1/2,117 (0.047%) |
| Total | 7/14,335 | 1/2,048 (0.05%) | 9/13,999 | 1/1,533 (0.064%) |

pregnancies without risk factors. The incidence of GBS EOS in term pregnancy was low even though patients with risk factors did not usually receive chemoprophylaxis in the absence of a defined protocol (Table 2).

During the 2nd period (1990–91), after the introduction of a policy of chemoprophylaxis, there were 9 cases of GBS EOS. One case involved a 24-week gestation in which the precipitous delivery precluded the administration of chemoprophylaxis during the intrapartum period. The other 8 cases of GBS EOS involved pregnancies of 36 weeks gestation or longer (Table 3). Three of the 8 had P-ROM (≥18 h) and received oxytocin induction of labor for this indication. P-ROM could not be predicted with certainty at the onset of labor, but could have been anticipated in these 3 cases after 12 h of ROM (patients 3, 5, and 6, Table 3). One case of GBS EOS was associated with ROM of 14 h duration in a patient undergoing induction of labor for post-dates pregnancy (patient 9, Table 3). Two patients at term developed fever during labor: patient 9 was treated with ampicillin and gentamicin 65 min prior to delivery and patient 6 had penicillin allergy and received cefoxitin and gentamicin 2 h prior to delivery (Table 3). Chemoprophylaxis is most effective if administered at least 4 h prior to delivery.[10]

In 1 case, an antepartum vaginal culture obtained 3 days prior to delivery was negative, although the neonate was heavily colonized at the time of delivery. The only risk factor in this case was preterm delivery at 36 weeks gestation (patient 2, Table 3).

One patient had spontaneous labor after spontaneous ROM and developed a fetal tachycardia. Maternal fever was detected during the cesarean section for fetal distress. This case represents the association of intrapartum fever with GBS EOS (patient 8, Table 3). Two patients (4 and 7, Table 3) had no risk factors.

The 3 neonatal deaths during the 4-year study occurred in preterm infants who did not receive chemoprophylaxis (Tables 2, 3).

### DISCUSSION

Randomized prospective studies have demonstrated that GBS EOS can be prevented by chemoprophylaxis of colonized, high-risk parturients.[2,3] The impact of selective chemoprophylaxis on the incidence of GBS EOS in a community hospital has not been described outside prospective clinical trials. Garland and Fliegner[11] achieved a reduction in GBS EOS by screening all their public patients at 32 weeks gestation and by treating every colonized parturient. A recent analysis demonstrated that universal prenatal screening for GBS and chemoprophylaxis of colonized women with labor complications are likely to be cost-beneficial in the United States.[12]

The best management strategy to prevent EOS GBS has been sought.[13] However, a complete consensus has not been achieved.[14] Recent guidelines from the American College of Obstetricians and Gynecologists (ACOG)[15] differ from those promulgated by the Committee on Infectious Diseases and the Committee on Fetus and Newborn.[10] Specifically, the ACOG regards screening for GBS as an option, whereas the pediatric group recommends screening of all pregnant women. The situation in which maternal GBS status is unknown and a risk factor for GBS EOS is present is addressed in a similar manner by both groups. The ACOG bulletin states that "it may be reasonable to use antibiotic chemoprophylaxis empirically." The pediatric rec-

TABLE 2. Data on cases of GBS EOS from the period before the introduction of a protocol for chemoprophylaxis of GBS EOS (1988–89)[a]

| Patient | Age (years) | G, P | Gestational age (weeks) | Risk factors | Intrapartum GBS culture | Chemoprophylaxis | Neonatal outcome |
|---|---|---|---|---|---|---|---|
| 1 | 32 | 7, 3 | 31.2 | Preterm P-PROM | Positive | None | Home, intact |
| 2 | 37 | 4, 3 | 32.5 | Preterm P-PROM | Not done | None | Died |
| 3 | 43 | 8, 4 | 34.2 | Preterm P-PROM | Positive | None | Home, intact |
| 4 | 34 | 2, 0 | 35.4 | Preterm P-PROM | Negative | None | Home, intact |
| 5 | 40 | 3, 1 | 35.7 | Preterm P-PROM | Not done | None | Home, intact |
| 6 | 32 | 3, 0 | 38.3 | None | Not done | None | Home, intact |
| 7 | 16 | 1, 0 | 39.0 | None | Not done | None | Home, intact |

[a]G = gravidity, P = parity; P-PROM = prolonged premature rupture of membranes (≥18 h).

ommendation for this situation is that "Chemoprophylaxis may be appropriate."

Although there is general agreement with the need for selective chemoprophylaxis, the feasibility and effectiveness of implementation have not been described in the community. A cost-benefit analysis by Strickland et al.[16] suggested that it would be cost-effective to provide chemoprophylaxis on the basis of a rapid test performed during labor. Their analysis assumed a very rapid turnaround time. It is not possible to reliably identify women who are GBS-colonized in the intrapartum period for 2 reasons. First, the diagnostic tests are not adequately reliable.[4,6,17] Second, implementing a rapid diagnostic test requires laboratory services around the clock, which is not feasible in many institutions.

Minkoff and Mead[7] recommended intrapartum treatment with ampicillin (2 g q 6 h for 24 h) of all mothers who deliver preterm infants and who are either carriers of group B beta-hemolytic streptococci or whose GBS carriage status is unknown. They[7] did not recommend treating term patients with P-ROM.

In our study, the incidence of GBS EOS in both time periods was lower than that described in other studies.[18] This may be related to the fact that pregnant women in the Los Angeles area and at this medical center have lower rates of GBS colonization (approximately 2.4–5.7%) than described elsewhere in the United States (Greenspoon, unpublished data).[17,19,20] The prevalence of GBS colonization was 22.8% of 5,586 pregnant women reported by Boyer et al.[19] and 18.6% of 7,742 pregnant women reported by Regan et al.[20] The low incidence of GBS EOS increases the risk of a beta error in a study of this size. A larger study is needed to have the power to demonstrate the statistical significance of small decreases in incidence rates.

In this study, chemoprophylaxis for preterm pregnancies at high risk for GBS EOS was administered more consistently during the 2nd period. There was a non-significant decrease in the number of cases among very preterm neonates. The implementation of an education program for chemoprophylaxis did not cause a statistically significant decrease in the rate of GBS EOS. The inability to demonstrate a benefit from chemoprophylaxis is due to the combination of the inadequate predictive value of antenatal cultures for intrapartum GBS colonization (patients 1 and 2, Table 3); non-compliance in administering indicated chemoprophylaxis (patients 3 and 5; Table 3); failure of chemoprophylaxis or intraamniotic infection treatment to prevent neonatal infection (patients 6, 8, and 9, Table 3); and the occurrence of GBS EOS in infants without risk factors (patients 4 and 7, Table 3).

Only 1 case of GBS EOS occurred in pregnancies of fewer than 36 weeks gestation during the 2nd time interval when chemoprophylaxis was recommended. This case involved a patient with a cervical cerclage who presented in active PTL at 24 weeks. She had a negative genital culture for GBS 5 weeks prior to admission. The patient delivered 25 min after arrival at the hospital. Boyer et al.[18] observed that delivery within 1 h of antibiotic therapy did not prevent GBS EOS. Thus, this patient would not have benefited from chemoprophylaxis because she delivered too rapidly (patient 1, Table 3).

The other cases of GBS EOS involved pregnan-

TABLE 3. Data on cases of GBS EOS from the period after the introduction of a protocol for chemoprophylaxis of GBS EOS (1990–91)[a]

| Patient | Age (years) | G, P | Gestational age (weeks) | Risk factors | Intrapartum GBS culture | Chemoprophylaxis or therapy | Neonatal outcome |
|---|---|---|---|---|---|---|---|
| 1 | 34 | 5, 2 | 24.0 | Preterm | Not done | None | Died |
| | | | | | Negative 5 weeks before delivery | | |
| 2 | 25 | 1, 0 | 36.0 | Preterm | Not done | None | Died |
| | | | | | Negative 3 days prior to delivery | | |
| 3 | 25 | 2, 0 | 36.6 | Preterm P PROM | Not done | None | Home, intact |
| 4 | 30 | 1, 0 | 37.1 | None | Not done | None | Home, intact |
| 5 | 31 | 1, 0 | 38.0 | P-PROM | Not done | None | Home, intact |
| 6 | 37 | 2, 1 | 39.0 | P-PROM, fever | Not done | Cefoxitin, gentamicin (penicillin allergy; 2 h prior to delivery) | Home, intact |
| 7 | 28 | 2, 1 | 39.2 | None | Not done | None | Home, intact |
| 8 | 33 | 2, 0 | 39.5 | Fever during cesarean section | Not done | None | Home, intact |
| 9 | 27 | 2, 1 | 41.5 | 14 h ROM, fever | Not done | Ampicillin, gentamicin (65 min prior to delivery) | Home, intact |

[a]G = gravidity, P = parity; P-PROM = prolonged premature rupture of membranes (≥18 h).

cies at or near term (36 or more completed menstrual weeks). Some of these cases had P-ROM. In all cases, it would have been possible after 12 h to prospectively determine that patients were likely to have P-ROM and to initiate chemoprophylaxis during labor (patients 3, 5, and 6, Table 3).

We emphasize the association of the induction of labor for PROM and GBS EOS in 3 of the 9 cases. Furthermore, a 4th case of GBS EOS was associated with ROM of 14 h duration in a patient undergoing induction of labor for post-dates pregnancy (patient 9, Table 3). Patients who require an induction of labor for spontaneous PROM may become candidates for chemoprophylaxis if they do not deliver within 18 h. It may be prudent to determine the GBS status of a patient who is likely to undergo induction of labor within a few days and whose induction of labor may be complicated by P-ROM.

Chemoprophylaxis is not of proven benefit in term pregnancy when the maternal GBS status is unknown and risk factors are absent. McDuffie et al.[21] reported adverse perinatal outcomes associated with resistant Enterobacteriaceae after treatment with ampicillin or amoxicillin in 4 mothers and their 5 infants. Further study of the potential adverse effects and their costs is justified. The incidence of GBS EOS in term pregnancy in both periods approaches the risk for fatal anaphylaxis due to penicillin therapy, which is approximately 1/10,000 cases.[22] None of the term infants in our study died or had ongoing disability at the time of discharge.

### CONCLUSIONS

Our experience indicates that a voluntary policy of chemoprophylaxis for GBS did not decrease the rate of GBS EOS. Even if compliance were 100%, cases of GBS EOS would continue to occur among some patients who become GBS-colonized after a negative antenatal GBS culture; among some patients in whom appropriate chemoprophylaxis or therapy for intraamniotic infection is ineffective; and among GBS-colonized patients without risk factors. One strategy to decrease the incidence of GBS EOS is to offer chemoprophylaxis to all colonized patients, including those without risk factors. This approach should receive careful cost-benefit and cost-effectiveness analysis before it is recommended. The adverse perinatal outcomes due to ampicillin-resistant microorganisms should be included in any risk-benefit analysis.[21] Thus, despite empiric chemoprophylaxis, GBS EOS will continue to occur, albeit infrequently, among parturients without risk factors.

### REFERENCES

1. Vollman JH, Smith WL, Ballard ET, Light IJ: Early onset group B streptococcal disease: Clinical, roentgenographic, and pathologic features. J Pediatr 89:199–203, 1976.

2. Boyer KM, Gadzala CA, Kelly PD, Gotoff SP: Selective intrapartum chemoprophylaxis of neonatal group B streptococcal early-onset disease. III. Interruption of mother-to-infant transmission. J Infect Dis 148:810–816, 1983.
3. Boyer KM, Gotoff SP: Prevention of early-onset neonatal group B streptococcal disease with selective intrapartum chemoprophylaxis. N Engl J Med 314:1665–1669, 1986.
4. Greenspoon JS, Wilcox JG, Kirschbaum TH: Group B *Streptococcus:* The effectiveness of screening and chemoprophylaxis. Obstet Gynecol Surv 46:499–508, 1991.
5. Anthony BF, Okada DM, Hobel CJ: Epidemiology of group B *Streptococcus:* Longitudinal observations during pregnancy. J Infect Dis 137:524–530, 1978.
6. Yancey MK, Armer T, Clark P, Duff P: Assessment of rapid identification tests for genital carriage of group B streptococci. Obstet Gynecol 80:1038–1047, 1992.
7. Minkoff H, Mead P: An obstetric approach to the prevention of early-onset group B beta-hemolytic streptococcal sepsis. Am J Obstet Gynecol 154:973–977, 1986.
8. Mead PB: When to treat intra-amniotic infection (editorial). Obstet Gynecol 72:935–936, 1988.
9. Gardner SB, Winter PD, Gardner MJ: Confidence Interval Analysis (CIA). Version 1.0. Br Med J 19–24, 1989.
10. Committee on Infectious Diseases and Committee on Fetus and Newborn: Guidelines for prevention of group B streptococcal (GBS) infection by chemoprophylaxis. Pediatrics 90:775–780, 1992.
11. Garland SM, Fliegner JR: Group B *Streptococcus* (GBS) and neonatal infections: The case for intrapartum prophylaxis. Aust NZ J Obstet Gynaecol 31:119–122, 1991.
12. Mohle-Boetani JC, Schuchat A, Plikaytis BD, Smith JD, Broome CV: Comparison of prevention strategies for neonatal group B streptococcal infection: A population-based economic analysis. JAMA 270:1442–1448, 1993.
13. Gibbs RS, Hall RT, Yow MD, McCracken GH Jr, Nelson JD: Consensus: Perinatal prophylaxis for group B streptococcal infection. Pediatr Infect Dis J 11:179–183, 1992.
14. Peter G, Lepow ML, McCracken GH, Phillips CF (eds): Group B Streptococcal Infections. Report of the Committee on Infectious Diseases. 22nd Ed. Elk Grove Village, IL: American Academy of Pediatrics, pp 447–450, 1991.
15. American College of Obstetrics and Gynecology: Group B Streptococcal Infections in Pregnancy. ACOG Technical Bulletin No. 170, pp 1–5, 1992.
16. Strickland DM, Yeomans ER, Hankins GDV: Cost-effectiveness of intrapartum screening and treatment for maternal group B streptococci colonization. Am J Obstet Gynecol 163:4–8, 1990.
17. Greenspoon JS, Fishman A, Wilcox JG, Greenspoon RL, Lewis W: Comparison of culture for group B *Streptococcus* versus enzyme immunoassay and latex agglutination rapid tests: Results in 250 patients during labor. Obstet Gynecol 77:97–100, 1991.
18. Boyer KM, Gadzala CA, Burd LI, Fisher DE, Paton JB, Gotoff SP: Selective intrapartum chemoprophylaxis of neonatal group B streptococcal early-onset disease. I. Epidemiologic rationale. J Infect Dis 148:795–801, 1983.
19. Boyer KM, Gadzala CA, Kelly PD, Burd LI, Gotoff SP: Selective intrapartum chemoprophylaxis of neonatal group B streptococcal early-onset disease. II. Predictive value of prenatal cultures. J Infect Dis 148:802–809, 1983.
20. Regan JA, Klebanoff MA, Nugent RP for the Vaginal Infection and Prematurity Study Group: The epidemiology of group B streptococcal colonization in pregnancy. Obstet Gynecol 77:604–610, 1991.
21. McDuffie RS, McGregor JA, Gibbs RS: Adverse perinatal outcome and resistant Enterobacteriaceae after antibiotic usage for premature rupture of the membranes and group B *Streptococcus* carriage. Obstet Gynecol 82:487–489, 1993.
22. Goodman LS, Gilman AG (eds): Goodman and Gilman's The Pharmacological Basis of Therapeutics. 8th Ed. New York: Pergamon Press, 1990.
